# Metal Transfer Behavior and Molten Pool Dynamics in Cold Metal Transfer Pulse Advanced Additive Manufacturing of 7075 Aluminum Alloy

**DOI:** 10.3390/mi15121489

**Published:** 2024-12-12

**Authors:** Yili Wang, Dongbin Zhang, Chen Zhang

**Affiliations:** 1 The Institute of Technological Sciences, Wuhan University, Wuhan 430072, China; yili_wang@whu.edu.cn; 2 Structural System Research Laboratory, Aerospace System Engineering Shanghai, Shanghai 201109, China; dongbin_zhang9505@163.com

**Keywords:** aluminum alloy, wire arc additive manufacturing, molten pool dynamics, numerical simulation

## Abstract

Wire arc additive manufacturing (WAAM) with a special arc mode of cold metal transfer pulse advanced (CMT-PADV) is an ideal additive manufacturing process for fabricating aerospace components, primarily high-strength aluminum alloys, offering advantages such as high deposition rates and low cost. However, the numerical simulation of the CMT-PADV WAAM process has not been researched until now. In this study, we first developed a three-dimensional fluid dynamics model for the CMT-PADV WAAM of 7075 aluminum alloy, aiming at analyzing the droplet transition and molten pool flow. The results indicate that, under the CMT-PADV mode, droplet transition follows a mixed transition mode, combining short-circuiting and spray transition. The Direct Current Electrode Positive period of the arc accelerates droplet spray transition, significantly increasing molten pool flow. In contrast, the Direct Current Electrode Negative period of the arc predominantly features droplet short-circuiting transition with low heat input and a weak impact on the molten pool. The periodic switching of the current polarity of CMT-PADV mode results in periodic variations in molten pool size and volume, reducing heat input while maintaining high deposition quality. The revelation of this mechanism provides process-based guidance for low-defect, high-performance manufacturing of critical components.

## 1. Introduction

The development of wire arc additive manufacturing of aluminum alloys is still in its infancy, mostly involving research on the material structure and static mechanical properties. As a difficult-to-weld material, aluminum alloys are highly susceptible to the formation of defects, including porosity and cracks, which represent significant challenges. At present, the mechanisms of defect formation and the methods of defect suppression are not yet well established in WAAM, but are closely related to the molten pool kinetic process. Therefore, there is an urgent need for research on heat transfer and fluid analysis in molten pools. However, experimental observation of the molten pool is very difficult and can only be achieved by synchrotron radiation high-speed imaging, X-ray high-speed imaging, and other extremely costly methods. Numerical simulation can display the physical data of molten pools more intuitively, which is very conducive to the study of the evolution law of molten pools.

At present, cold metal transfer (CMT) technology is the most widely used arc heat source for WAAM. The CMT technology has been developed by Fronius since 2004, and through the continuous efforts of the technology developers, new modes have been introduced based on the CMT technology, such as the CMT Pulse mode, the CMT Advanced (CMT-ADV) mode, the CMT Pulse Advanced (CMT-PADV) mode, etc. As a new technology, the torch in CMT Pulse mode can provide a periodic pulse current to quickly melt the wire into a molten droplet, with the molten droplet released from the wire into the molten pool by electromagnetic force. The droplet transition in CMT Pulse mode is the combination of a spray transition during each pulse current period and a short-circuiting transition during the short-circuiting period. The CMT-ADV mode is based on the conventional CMT technology and introduces a polarity transition. The CMT-PADV mode was developed based on CMT Pulse mode and CMT-ADV mode. The current cycle in CMT-PADV mode can be divided into the Direct Current Electrode Positive (DCEP) period and the Direct Current Electrode Negative (DCEN) period according to the current polarity, with the positive period being the spray transition and the negative period being the conventional short-circuiting transition. Cong et al. [1]. investigated the impact of four arc modes, namely conventional CMT, CMT-Pulse, CMT-ADV and CMT-PADV, on the porosity of the AA2219 alloy in WAAM. The findings indicated that the conventional CMT mode is the most susceptible to porosity, while the CMT-PADV mode is the most effective in reducing porosity. Consequently, it is imperative to conduct a simulation study of the droplet transition and molten pool dynamics for CMT-PADV WAAM.

The current numerical models achieve the simulation of the droplet transition and molten pool of the CMT-WAAM process. Zhao et al. [2] established a more comprehensive model of droplet transition in CMT mode, which realized the simulation of the wire movement process of “arc ignition–short circuit–retraction–transition”, and proved that the periodic retraction of the wire during the welding process will trigger the periodic oscillation of the molten pool and improve the welding quality. Han et al. [3] carried out a study on the molten pool dynamics and the effect of different driving forces on the molten pool in the CMT current cycle. The findings revealed that the temperature field and flow field distribution of the molten pool at the moment of peak current and base current in CMT mode are almost unchanged, but the maximum flow rate of the molten pool at the moment of peak current is significantly increased. The primary driving force of the flow field in molten pool has been identified as the electromagnetic force. Lin et al. [4] employed a three-dimensional dynamic numerical model to investigate the spreadability of AA4043 alloy drops on Q235 steel under disparate welding conditions. The simulation results demonstrated that the most critical factor influencing the spreadability of droplets is temperature. The solidification of droplets at the three-phase line impedes the spreading of droplets. As the wire feeding speed increases, the temperature of the molten pool and the temperature gradient at the edge of molten pool become more pronounced, leading to an enhancement of the Marangoni force and droplet spreadability. Azar et al. [5] incorporated the oscillatory behavior of the current in CMT welding into their dual ellipsoid heat source model, thereby enabling the heat flux density and heat source range to vary in accordance with the current. This study investigated the periodic variation in the molten pool and the effect of short-circuiting transition on the molten pool at a time-step scale of 1 ms. It is demonstrated that the expansion of the heat source range of the molten pool during the peak current phase is driven by the heat flux at the cost of lowering the heat flux density. However, the simulations are limited by the time to solution, which restricts the calculation to a single current cycle. Additionally, Zapico et al. [6] employed a transient dual ellipsoid heat source model to simulate the welding process on a 3 mm thick AA5754 alloy sheet, subsequently verifying the model through the utilization of thermocouple temperature measurements. The considerable discrepancy between the peak and average temperatures observed in the simulation illustrates the CMT’s capacity to dynamically adjust the current in order to enhance the quality of the deposition.

It can be concluded from the above that the simulation study of WAAM is dominated by the CMT mode, while the simulation study of other arc modes is relatively rare. Given the unparalleled advantage of CMT-PADV in WAAM of aluminum alloys, it is imperative to investigate the dynamic evolution of the molten pool in CMT-PADV mode, which will provide a means of analysis for the mechanism exploration and subsequent process optimization.

The primary aim of this study is to thoroughly investigate the effects of the droplet transition, the interaction between droplets and molten pool, and the current polarity on the characteristics of the molten pool and the forming stability in CMT-PADV mode. First, we established a 3D thermal–fluid field coupled numerical model of WAAM in CMT-PADV mode. The most significant aspect of the model is its incorporation of the effects of current polarity and the type of droplet transition on the characteristics of the molten pool in CMT-PADV mode, which represents a significant innovation. The effect of the sharp impact on the molten pool caused by the spray transition during the pulse phase is discussed. Combined with high-speed photography, the dynamic evolution mechanism of the molten pool during deposition in the CMT-PADV mode is revealed.

## 2. Experimental Procedure and Analysis Methods

### 2.1. Experimental Set-Up and Procedure

**Figure 1:**
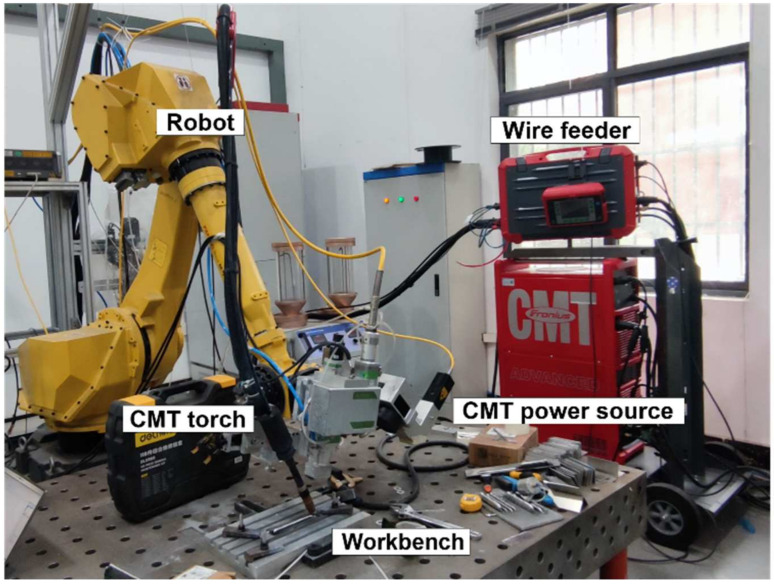
CMT-WAAM experimental system.

In this study, the CMT-WAAM experimental system was used, as shown in Figure 1, which consists of a CMT welding machine, a wire feeder, a welding torch, an industrial robot, and a workbench. The motion control was performed by a M-710iC/50L industrial robot (FANUC Corporation, Minamitsuru, Japan). The welding apparatus employed was the TPS 4000 CMT welding machine (Fronius International GmbH, Vienna, Austria), which is capable of providing four droplet transition modes: conventional CMT, CMT Pulse, CMT ADV, and CMT-PADV. A 7075 aluminum alloy welding wire (Longshuo Welding Materials Co., Ltd., Shanghai, China) with a diameter of 1.2 mm was used for additive manufacturing, and the substrate was a 1060 aluminum plate with a dimension of 200 × 150 × 15 mm (Wuhan Xinghuan Aluminum Industry Co., Ltd., Wuhan, China). The specific composition of the above is detailed in Table 1. The shielding gas employed in the experiment was high-purity argon (99.99%). In accordance with the preliminary process window exploration, the process parameters employed in the experiment are presented in Table 2.

**Figure 2:**
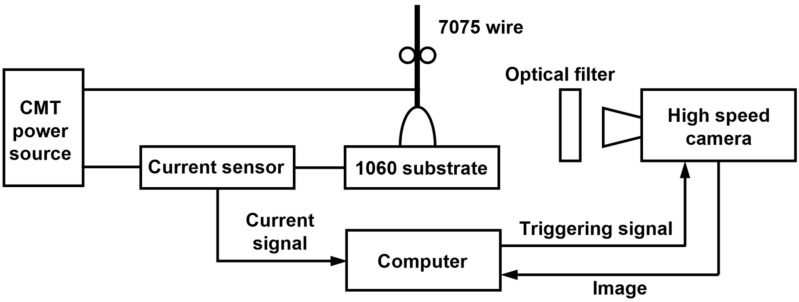
Schematic of high-speed photographic signal acquisition system.

**Figure 3:**
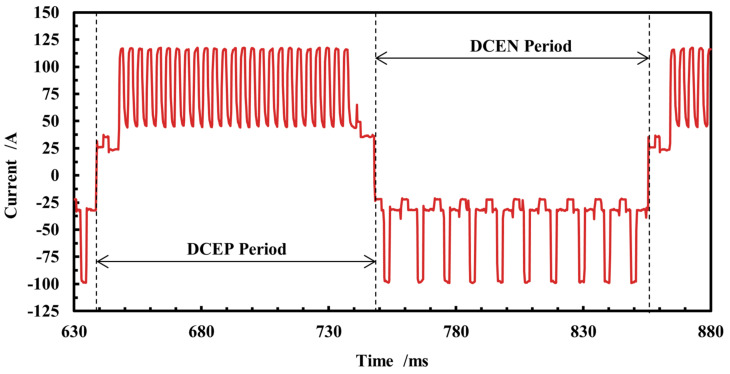
Arc current waveform in CMT-PADV mode.

The high-speed camera signal acquisition system used to capture the droplet transition and current pulse waveforms during the deposition process is shown in Figure 2. The high-speed camera is an i-SPEED 726 (iX Cameras Ltd., Essex, UK) with a frame rate of 5000 fps. The light intensity is reduced by a light filter to improve the image quality. The current waveform is captured by the sensor and transferred to the computer, where the control software ensures the synchronous operation and matching of the image and current acquisition system. As illustrated in Figure 3, the duration of the CMT-PADV current waveform is approximately 220 ms, comprising 25 positive polarity spray transitions, 10 negative polarity short-circuiting transitions, and 2 current polarity changes. Notably, the two cycles of DCEP and DCEN are of equal lengths, with a duration of 110 ms each.

### 2.2. Mathematical Model and Numerical Simulation

In this paper, the molten metal is assumed as incompressible flow, laminar flow, and Newtonian fluid.

#### 2.2.1. Governing Equations

The mass transfer and heat transfer in the molten pool in CFD can be calculated from the equations for conservation of mass, momentum, and energy. The equations are expressed as follows:

Mass:

(1) $$\frac{\partial \rho }{\partial t}+\nabla \cdot (\rho \cdot \vec{v})=0$$

where *ρ* is the density, *t* is the time, and $\vec{v}$ is the velocity vector.

Momentum:

(2) $$\rho (\frac{\partial \vec{v}}{\partial t}+\vec{v}\cdot \nabla \vec{v})=-\nabla p+\mu \nabla^{2}\vec{v}-\frac{\mu \vec{v}}{K}+\rho \vec{g}\beta (T-T_{ref})+\vec{F}$$

where *p* is the pressure, μ is the viscosity, and K is the drag coefficient in the porous media; $\vec{g}$ is the gravitational vector, $T_{ref}$ is the reference temperature, and $\vec{F}$ is the electromagnetic force vector.

Energy:

(3) $$\frac{\partial (\rho H)}{\partial t}+\vec{v}\cdot \nabla (\rho H)=\nabla \cdot (k\nabla T)+S_{V}$$

where *H* is the enthalpy; *k* is the thermal conductivity; $S_{V}$ is the source term.

In this paper, the VOF method [7] and enthalpy–porosity technique [8] are adopted to track the gas–metal free interface and the solid–liquid interface, respectively.

#### 2.2.2. Models of Heat Source and Driving Forces in CMT-PADV-WAAM

The dual ellipsoidal heat source model is used to describe the heat flux distribution of an arc, as described in the following equations:

(4) $$q(x,y,z)=\frac{6\sqrt{3}f_{1}\eta UI}{\pi a_{f}bc\sqrt{\pi }}\cdot \exp(-3\frac{x^{2}}{{a_{f}}^{2}})\cdot \exp(-3\frac{y^{2}}{b^{2}})\cdot \exp(-3\frac{z^{2}}{c^{2}}),x>0$$

(5) $$q(x,y,z)=\frac{6\sqrt{3}f_{2}\eta UI}{\pi a_{r}bc\sqrt{\pi }}\cdot \exp(-3\frac{x^{2}}{{a_{r}}^{2}})\cdot \exp(-3\frac{y^{2}}{b^{2}})\cdot \exp(-3\frac{z^{2}}{c^{2}}),x\leq 0$$

where *η* is the thermal effect coefficient of the arc; *U* is the arc voltage; *I* is the arc current; $f_{1}$ and $f_{2}$ are the distribution coefficients of the ellipsoidal heat source before and after the x-axis; $a_{f}$, $a_{r}$ are the shape parameters of the ellipsoidal heat source in the x-axisbefore and after direction. *b* is the shape parameter in the y-axis direction, *c* is the shape parameter in the z-axis direction. In this work, $a_{f}$ and $a_{r}$ are taken as 0.002 and 0.004, *b* as 0.002, *c* as 0.003, $f_{1}$ and $f_{2}$ as 2/3 and 4/3, respectively, and *η* as 0.85 [9].

The calculation also considered the main force sources acting on the molten pool, such as arc pressure, arc shear force, droplet impact force, electromagnetic force, gravity, buoyancy, and surface tension. The driving force expressions and material thermo-physical properties have been detailed in previous studies [10].

#### 2.2.3. Computational Domain and Boundary Conditions

**Figure 4:**
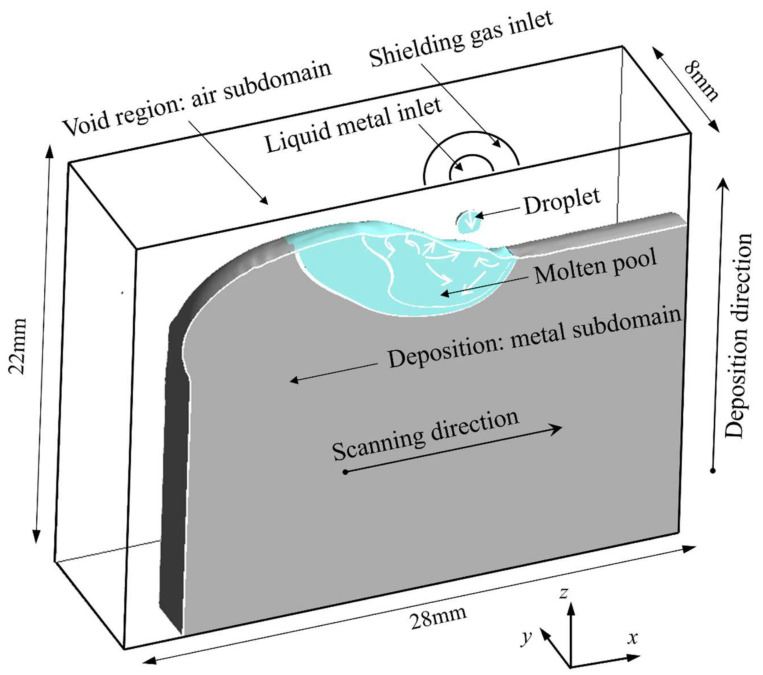
Schematic of solution domain.

The computational model is shown in Figure 4, with a size of 28 mm × 8 mm × 22 mm. The meshes are hexahedral meshes, in which the scanning area of the molten pool is an encrypted area with a mesh edge length of 0.2 mm, and the rest of the area has a mesh edge length of 0.4 mm. The solid part of the model is the deposited layer and the molten pool, and the void area is the shielding gas. The top of the model is the velocity inlet, from which the molten metal enters the molten pool to simulate the droplet transition. The arc heat source is used as part of the heat flow boundary conditions on the free surface of the molten pool, and the main modes of energy transfer on the free surface of the molten pool are convection, radiation, and evaporation, which can be expressed as follows [11]:

(6) $$-k\frac{\partial T}{\partial \vec{n}}=q_{arc}-\alpha (T-T_{0})-\varepsilon \sigma (T^{4}-T_{0}^{4})-m_{er}L_{b}$$

where $q_{arc}$ is the heat input from the arc; $\vec{n}$ is the vector unit normal to the workbench; α is the convective heat transfer coefficient; ε is the surface radiation emissivity; σ is the Stefan–Boltzmann constant; $m_{er}$ is the evaporation rate; $L_{b}$ is the latent heat of evaporation.

For other surfaces, only convection and radiation are considered:

(7) $$-k\frac{\partial T}{\partial \vec{n}}=q_{arc}-\alpha (T-T_{0})-\varepsilon \sigma (T^{4}-T_{0}^{4})$$

## 3. Results and Discussion

### 3.1. Droplet Transition in DCEP Period of CMT-PADV

**Figure 5:**
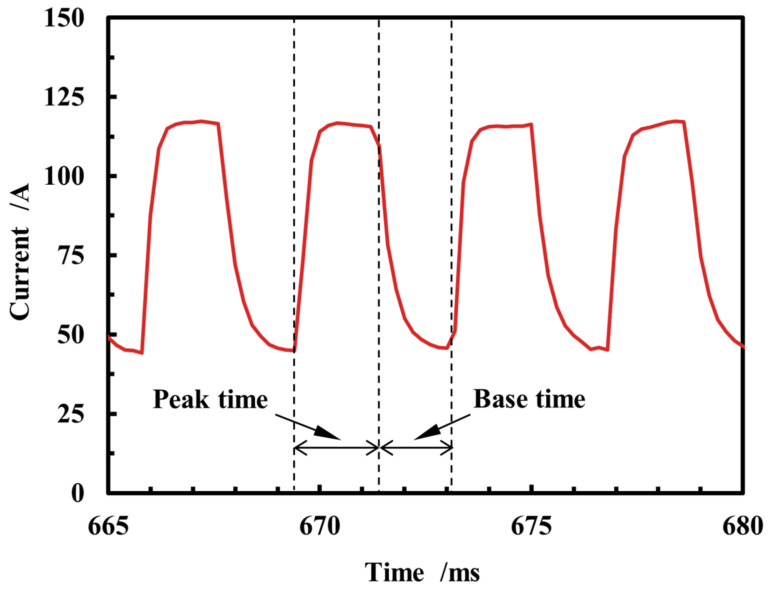
Current waveform of spray transition in DCEP period.

As illustrated in Figure 5, spray transition in the DCEP period can be delineated into a peak time and a base time. The peak current value is 116.6 A, with a duration of 2 ms, while the base current size is 45.8 A, with a duration of 1.8 ms.

**Figure 6:**
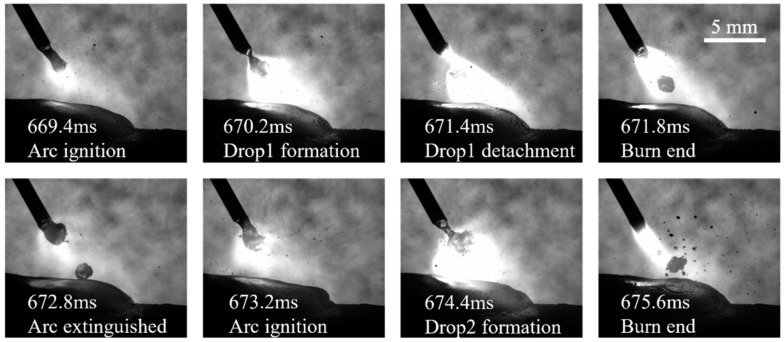
The spray transition during the CMT-PADV process.

Figure 6 illustrates the process of droplet transition in the pulse phase, which is characteristic of the “one pulse current, one droplet transition” phenomenon of spray transition. The current increases gradually during the arc ignition stage, resulting in the rapid melting of the wire into a molten droplet through the action of the current. Then, as the current attains its peak value, the molten droplet increases in size and gradually overcomes the surface tension that binds it to the molten pool. After that, the molten droplet is propelled by electromagnetic force away from the wire and towards the molten pool. As the droplet leaves the wire, the current enters a gradual decline, the arc light diminishes, and the wire tip begins to melt, preparing for the next spray transition. This marks the completion of a pulse current cycle.

When the current enters the arc ignition stage and starts the next cycle of droplet transition, the droplet from the previous transition cycle starts to enter the molten pool. From 672.8 ms to 675.6 ms, it can be observed that the droplet enters the molten pool and exerts a significant impact. This causes the interface to spring back to the surface and rise up under the action of the fluid, and the interface of the molten pool thus generates oscillations. When the surface state of the molten pool returns to the pre-impact state, the next droplet transition has already arrived, and the molten pool starts the next cycle of oscillation. Consequently, the flow change of the molten pool during the pulse phase is markedly more frequent and pronounced in comparison to the CMT mode.

**Figure 7:**
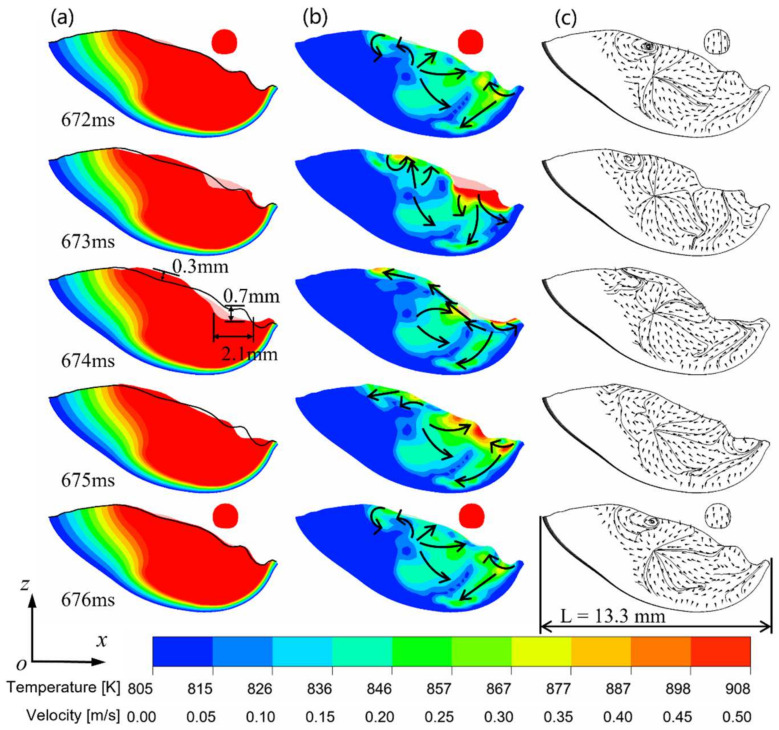
Morphology of the molten pool during spray transition in CMT-PADV mode (along the scanning direction): (**a**) temperature field; (**b**) velocity field; (**c**) streamline.

Figure 7 illustrates the alterations of the molten pool before and after the molten droplet enters the molten pool in the pulse phase. Figure 7a shows that the surface of the molten pool is always in a state of oscillation during the pulse phase of the DCEP period, due to the high frequency of droplet transition. A comparison of the morphology of the molten pool at 672 ms and 674 ms reveals the creation of a surface depression with a length of 2.1 mm and a depth of 0.7 mm in the center of the molten pool due to the impact of the droplet, while the rear of the molten pool exhibits an increase in height of approximately 0.3 mm. Combined with Figure 7b,c, the impact of the droplet on the molten pool is predominantly concentrated in the surface of the molten pool, as well as in the front and central portions of the pool.

At 672 ms, the molten droplet has not yet entered the molten pool, and the fluid flow exhibits a tendency towards the center of the pool, while a vortex exists in the rear of the molten pool surface. Additionally, the fluid flow in the front of molten pool surface is observed to be directed towards the center of molten pool, and the fluid at the center of the molten pool flows downwards from the surface to the bottom of the molten pool. At 673 ms, the droplet comes into contact with the surface of the molten pool, and the free surface fluid flow in the front of the pool changes to flow towards the edge of the pool; the fluid flow in the middle of the pool flows towards the bottom, while the surface fluid flow in the rear of the pool changes to flow upwards. At 674 ms, the droplet impact causes a surface depression in the molten pool, and the free surface flow in the front of molten pool continues to move towards the boundary, while the fluid flow at the center of the molten pool surface moves to the rear of the molten pool. At 675 ms, as the molten flow backfills the surface depression, the free surface flow near the center of the molten pool changes to a reflow from the front/rear of the molten pool to the center of the molten pool.

With the generation of the next pulse droplet, the flow distribution of the molten pool at 676 ms is restored to what it was before the droplet impact.

**Figure 8:**
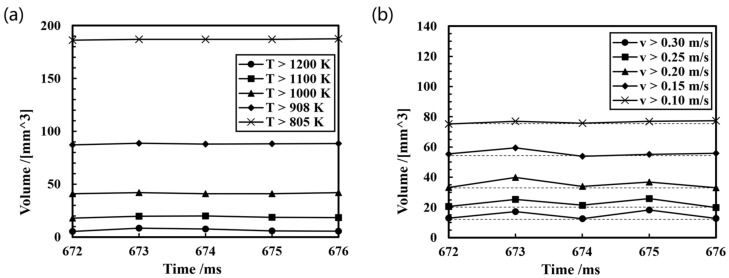
Trend of molten pool characteristics during the spray transition: (**a**) temperature field; (**b**) velocity field.

Figure 8 illustrates the trend of changes in the volume of the molten pool within various temperature and velocity ranges during a pulse cycle. Due to the brief pulse cycle time of 3.8 ms at a wire feeding speed of 8 m/min and a travel speed of 10 mm/s, the heating effect of the heat source on the molten pool is not substantial. From Figure 8a, it can be seen that the volume curves of the molten pool at various temperature ranges within one pulse cycle do not show significant changes. This is because the volume of one spray droplet is only 1.7 mm^3^, while the volume of the molten pool at various temperature ranges is relatively large, especially above the solidus temperature, which reaches 187 mm^3^. Consequently, the temperature field of the molten pool exhibits minor variations during a pulse cycle.

On the contrary, there is a significant change in the volume curves of the molten pool at various velocity ranges within one pulse cycle. At 673 ms, the volume of the molten pool with a velocity exceeding 0.3 m/s is increased by 4.7 mm^3^ relative to that at 672 ms, due to the impact of the spray droplet, which is evident in the pronounced expansion of the red region depicted in Figure 7b. As the surface depression caused by the droplet reaches its maximum, part of the kinetic energy of the droplet is converted into the internal potential energy of the molten pool, including surface energy, elastic potential energy, etc., and thus the volume of the molten pool decreases in all velocity ranges at 674 ms. At 675 ms, the fluid in the center of the molten pool begins to flow upward, resulting in a reduction in the surface deformation of the molten pool. This is accompanied by a release of potential energy within the molten pool, which is transformed into kinetic energy. So the volume of the molten pool in all velocity ranges increases by approximately 4 mm^3^, with the greatest volume increment observed in the velocity range exceeding 0.3 m/s, reaching 4.7 mm^3^. In combination, as the pool surface exhibits violent oscillations, the velocity magnitude of the flow in the molten pool undergoes periodic fluctuations during a pulse cycle.

**Figure 9:**
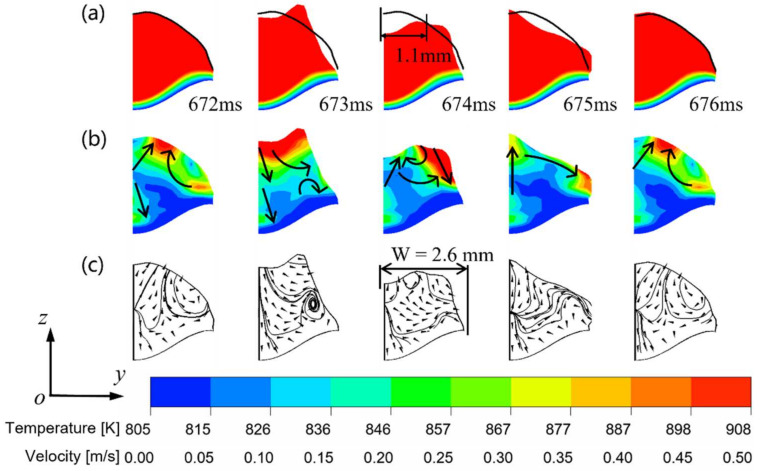
Morphology of the molten pool during spray transition in CMT-PADV mode (along the weld width direction): (**a**) temperature field; (**b**) velocity field; (**c**) streamline.

As illustrated in Figure 7a and Figure 9a, the free surface oscillation amplitude in the direction of the width of the molten pool is larger than in the direction of the length, because the width of the molten pool is small compared with the length of the molten pool, which results in a smaller buffer for the impact of the molten drop. From the flow field distribution in Figure 9b,c, it can be discerned that the primary alteration in the flow field of the molten pool occurs on the surface of the molten pool, while the direction of the flow field at the bottom of the molten pool, barely affected by the droplet, remains downward before and after the droplet drops.

At 672 ms, the flow direction on the molten pool surface is typically upward. At 673 ms, following the contact of the molten droplet with the molten pool, a notable increase in the red area on the surface of the molten pool, indicative of a flow velocity exceeding 0.5 m/s, is observed, while the direction of the surface fluid flow shifts to the side edge of the molten pool, forming a vortex at the bottom of the side edge. At 674 ms, the droplet initiates a depression with a radius of 1.1 mm on the molten pool surface. At 674 ms, the direction of the fluid flow near the depression is directed towards the center of the depressed surface, with the tendency of filling the depression. On the surface of the molten pool, the red color region, which represents a high flow rate, changes to flow to the outside of the molten pool along the surface. After the surface depression is filled with fluid, the direction of the fluid flow at the center of the molten pool becomes vertically upward, and the flow direction on the side of the surface still flows toward the outside of the molten pool. At 676 ms, the next pulse drop is generated and the flow field of the molten pool is restored to the same as at 672 ms.

### 3.2. Droplet Transition in DCEN Period of CMT-PADV

**Figure 10:**
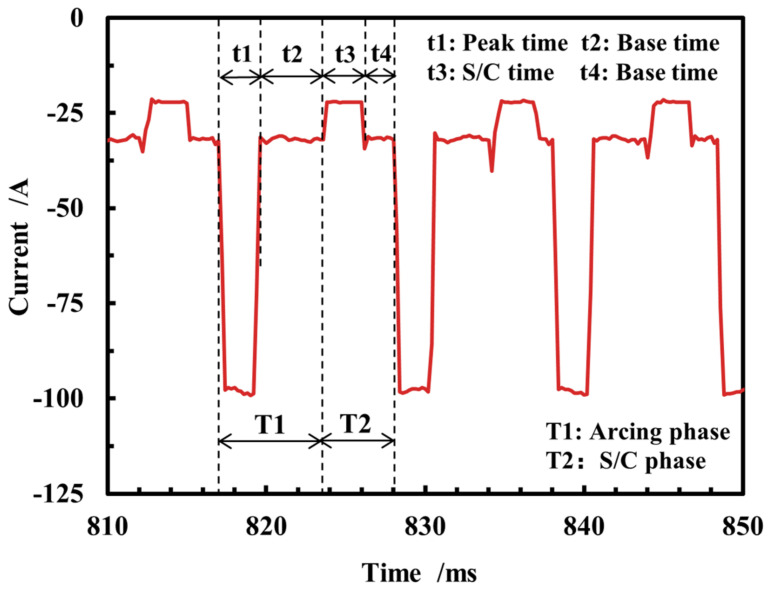
Current waveform of short-circuiting transition in DCEN period.

The current waveform during the short-circuiting transition in the DCEN period is shown in Figure 10. The DCEN short-circuiting transition can be divided into an arcing phase and a short-circuiting phase, and the arcing phase can be subdivided into a peak time and a base time.

In the DCEN period, the cathode spot, which was initially situated on the molten pool surface in the DCEP period and served to heat the molten pool, shifted to the wire tip due to the change in current polarity. This results in a reduction in the size of the molten pool, due to the diminished heat input brought by the cathode spot. Conversely, the droplet size in the wire experiences an increase, due to the increase in heat input and the enhanced coating rate. As the size of the droplet increases, the fluctuation of the droplet surface increases and the arc length changes frequently, which may lead to arc instability and bouncing between the wire and the molten pool, resulting in an unstable droplet transition [12]. Accordingly, when the droplet transition occurs in the DCEN period, in comparison to the DCEP period, the arc will be more unstable and the molten pool temperature and fluidity will be lower. Moreover, the low surface temperature in the DCEN period makes it difficult for the shielding gas to cover the arc area due to insufficient temperature and flow obstruction, and the protective effect is insufficient, resulting in an unstable arc and difficult arc ignition. Therefore, the reason why the DCEN cycle requires an additional base current stage of 1.8 ms in the short-circuiting phase is to ensure that the arc ignition of the next cycle runs smoothly by increasing the heat input and reducing the current magnitude fluctuations.

**Figure 11:**
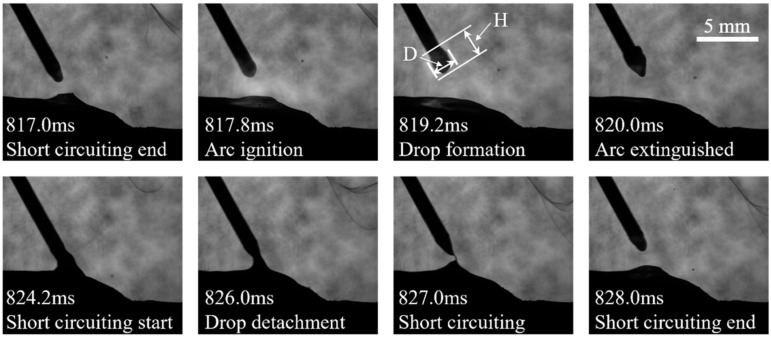
The short-circuiting transition during the CMT-PADV process.

The short-circuiting transition process in the DCEN period is shown in Figure 11. At 827 ms, the wire is separated from the molten pool surface during the base time of the short-circuiting phase. As presented in 817.8 ms and 819.2 ms, the arc wandering phenomenon occurs on the wire tip in the DCEN period. This phenomenon results from the transfer of the cathode spot from the molten pool to the wire tip, which increases the diameter of the droplets formed by the molten wire. This, in turn, prompts the arc to wander along the wire from the tip of the wire. In addition, arc instability caused by insufficient shielding gas protection under the DCEN cycle can also cause the arc wandering phenomenon [13].

As shown in Table 3 and Table 4, while the mean current of 83.1 A in the DCEP period is considerably higher than that in the DCEN period, the size of the droplets produced in the DCEN period is larger than that in the DCEP period. As previously stated, in the CMT-PADV mode, as the current polarity transitions from positive to negative, the cathodic spot that initially heated the molten pool shifts to the tip of the wire, which means that the heat input to melt the wire is significantly increased, and the short-circuit transition cycle is 7.2 ms longer than the pulse cycle, enabling the wire to melt and produce droplets for a longer time. This results in the formation of larger droplets during the DCEN period.

### 3.3. Evolution of Molten Pool in CMT-PADV Mode

**Figure 12:**
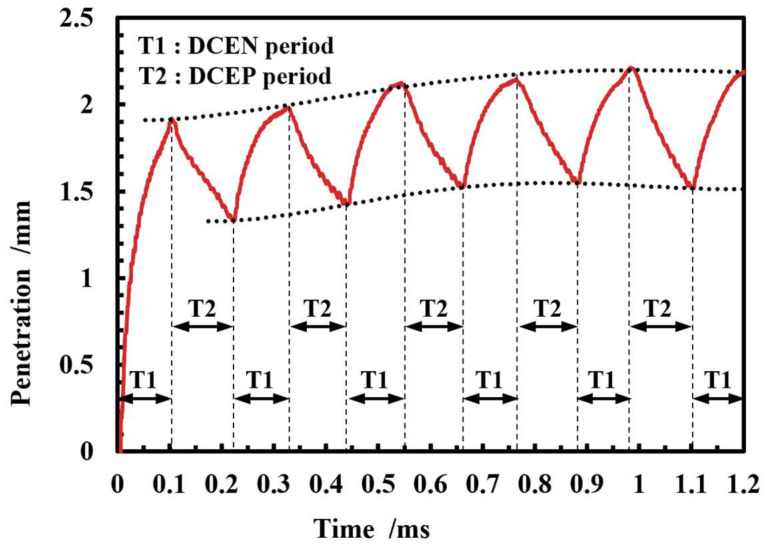
Variation in molten pool depth in CMT-PADV mode.

**Figure 13:**
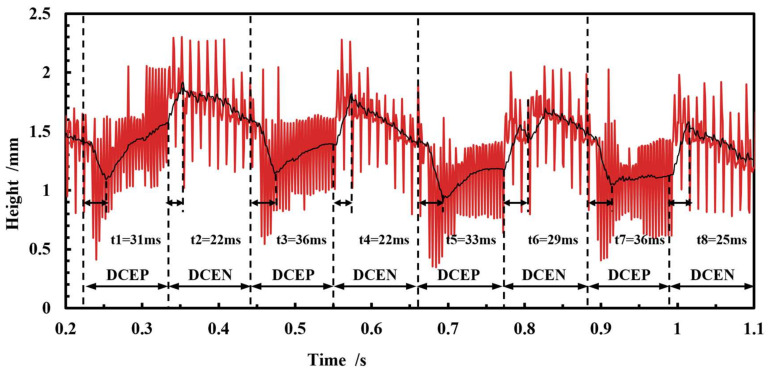
Variation in free surface height of the molten pool in CMT-PADV mode.

The variation in the molten pool depth and free surface height is shown in Figure 12 and Figure 13. The molten pool depth changes cyclically with the current polarity: the molten pool depth increases in the DCEP cycle and decreases in the DCEN cycle due to the decrease in the heat input to the molten pool brought about by the cathode spot shift. The overall trend of the molten pool depth is towards an increase. After 0.55 s, the molten pool depths at the end of the DCEP and DCEN cycles stabilized at 2.12 mm and 1.52 mm, respectively, and the difference between the DCEP and DCEN cycles reached a maximum of 0.68 mm at 0.99–1.1 s.

**Figure 14:**
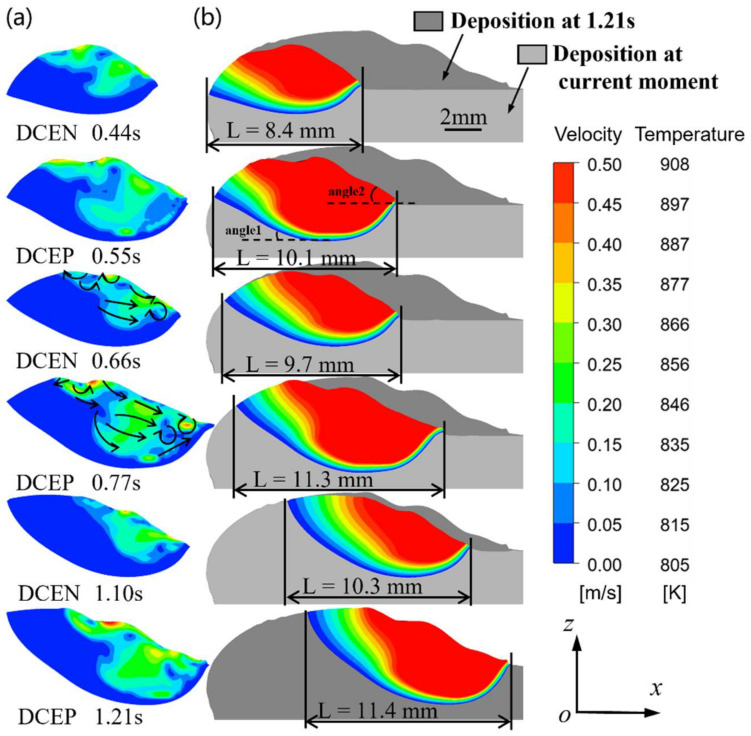
Evolution of molten pool characteristics in CMT-PADV mode: (**a**) velocity field; (**b**) temperature field.

The free surface height of the molten pool exhibits a cyclical variation in conjunction with the droplet transition over a relatively short duration. Similarly to the molten pool depth, the surface height of molten pool exhibits an increase during the DCEP period and a decrease during the DCEN period. During the DCEP period, the continuous impact of the droplets on the molten pool causes surface fluid flow to emerge from the center and extend towards the rear of the molten pool. This moves the droplet and fluid in the front of molten pool towards the rear of molten pool, increasing the pool surface height. During the DCEN period, as the molten pool and the droplet transition’s impact reduce, the fluid at the high point, located in the rear of the molten pool, flows gradually to the lower fluid located in front of the molten pool. Consequently, the surface height of the molten pool decreases gradually in the DCEN period. It is important to note that the impact of spray droplets on the melt pool does not immediately cease at the conclusion of the DCEP period. Consequently, a period of 22–29 ms of increased pool surface height will be observed at the outset of the DCEN period. Similarly, following the commencement of the DCEP period, the surface height of the molten pool will continue to decrease for a brief period, approximately 31-36 ms. As the deposition process continues, the molten pool undergoes a gradual expansion, accompanied by a concomitant decrease in the angle2 of the surface in the front of the pool, as shown in Figure 14b. This results in an overall reduction in the surface height, about 0.3 mm, during the period of 0.3–1.1 s.

**Figure 15:**
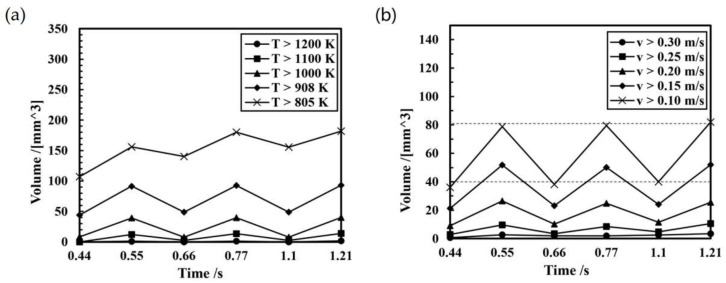
Trend of molten pool characteristics during the deposition process: (**a**) temperature field; (**b**) velocity field.

Figure 14 illustrates the variation in molten pool morphology, temperature field, and velocity field with time in the CMT-PADV mode, and the volume data of the molten pool at various temperature ranges and velocity ranges at the corresponding moments are shown in Figure 15. As shown in Figure 14b, as the deposition process continues, it becomes clear that the angle1 in the rear of the molten pool gradually increases, while the angle2 in the front surface of the molten pool gradually decreases, and the morphology of the molten pool remains relatively unchanged after 1.1 s.

As illustrated in Figure 14b and Figure 15a, the molten pool dimensions exhibited an increasing trend in general. At 0.55 s, the molten pool volume was recorded at 156.2 mm^3^, while at 0.77 s, it grew to 179.1 mm^3^. Subsequently, the molten pool reached the thermal equilibrium, resulting in minimal growth in both the length and volume of the molten pool, 0.1 mm and 2.8 mm^3^ respectively, in 0.77–1.21 s. The volume of the solid–liquid mushy zone (the molten pool fluid at a temperature of 805–908 K) during the period of 0.44–1.21 s was 64.7 mm^3^ (at 0.44 ms), 63.2 mm^3^ (at 0.55 ms), 91.4 mm^3^ (at 0.66 ms), 87.5 mm^3^ (at 0.77 ms), 107.4 mm^3^ (at 1.10 ms), and 88.9 mm^3^ (at 1.21 ms). Combined with the trend of the molten pool volume in Figure 15a, it can be seen that, during the DCEP period, the range of the molten pool increases and the range of the solid–liquid mushy zone in the molten pool decreases. In contrast, during the DCEN period, the size of the molten pool decreases, whereas the mushy zone of the molten pool increases. Overall, the molten pool morphology and flow field distribution are similar in the DCEP/DCEN period of different current cycles.

Analyzing the changes in the velocity field of the molten pool in Figure 14a and Figure 15b, it can be observed that the flow velocity of the molten pool increases in the DCEP period and decreases in the DCEN period. Furthermore, the distributions of flow direction in the DCEP/DCEN period of different current cycles are found to be analogous, and after 0.55 s, the volume data of the molten pool at all velocity ranges remain largely unaltered in the DCEP/DCEN period of different current cycles.

The dynamic evolution of the molten pool, with the DCEP and DCEN period continuously converted, allows the molten pool size to change constantly, and the molten pool temperature also experiences a “hot–cold” continuous cycle process, which facilitates the transfer of heat accumulated in the molten pool under the higher heat source power in the DCEP period to the substrate through the deposition layer during the DCEN period. This avoids the issue of the molten pool collapsing due to the continuous accumulation of excessive heat input in the molten pool, thereby ensuring the forming stability and enhancing the quality of the deposition layer.

**Figure 16:**
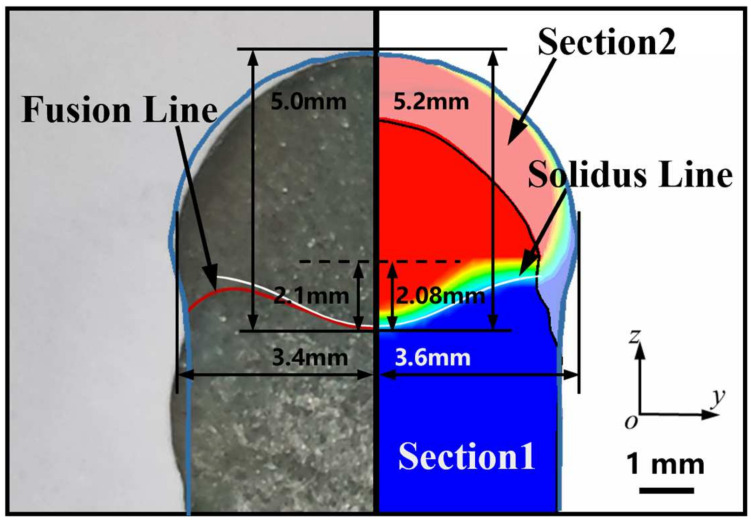
Comparison of experimental sample and simulation result (Sections 1 and 2 are located at the maximum of molten depth and layer height, respectively, at 1.21 s).

Figure 16 illustrates the comparison between the experimental sample and the simulation under identical process parameters. It can be observed that the discrepancy between the simulation and the sample in terms of profile and fusion line is insignificant, thereby substantiating the efficacy of the three-dimensional thermal–fluid field-coupled numerical model of WAAM in CMT-PADV mode.

## 4. Conclusions

(1) A 3D thermal–fluid field-coupled numerical model of WAAM in CMT-PADV mode was developed.
(2) The droplet transition in CMT-PADV mode is a mixed transition, combining the short-circuiting transition and the spray transition. The current cycle can be divided into the DCEP period and the DCEN period according to current polarity. In the DCEP period, the spray droplet induced a notable depression in the center of the molten pool, and the molten pool flow rate and direction fluctuated periodically in accordance with the magnitude of surface deformation of the molten pool. The transfer of the cathode spot to the wire in the DCEN period resulted in an increase in the amount of melting, consequently leading to a larger diameter for the short-circuiting droplet transition than that of the spray transition.
(3) In the DCEP period, the arc intensity increased significantly, accompanied by a notable expansion in the volume and flow rate of the molten pool. In addition, the extent of the solid–liquid mushy zone diminished, while the flow distribution in the molten pool evinced a periodic alteration, and the molten pool surface was perpetually in an oscillating state. In the DCEN period, the current gained negative polarity; the arc exhibited diminished intensity; the arc wandering phenomenon was observed at the tip of the wire; and the volume and flow rate of the molten pool decreased considerably, while the extent of the solid–liquid mushy zone increased significantly.
(4) During the deposition process, the volume, depth, and surface height of the molten pool center all increased in the DCEP period and decreased in the DCEN period, in which the shift in the trend of the surface height change was somewhat delayed in comparison with the shift in the current polarity. The cyclic change in molten pool characteristics with current polarity was conducive to reducing the heat input accumulation effect and improving the forming quality.

## Figures and Tables

**Table 1:** Chemical composition of the filler wire and substrate (wt %).

Material	Cu	Mg	Mn	Si	Fe	Ti	Zn	Cr	Al
7075	1.2–2.0	2.1–2.9	0.30	0.40	0.50	0.20	5.1–6.1	0.18	Bal
1060	0.05	0.03	0.03	0.25	0.35	0.03	0.05	-	Bal

**Table 2:** Deposition parameters used in experiments.

Parameter	Value	Unit
Wire feeding speed	8	m/min
Travel speed	10	mm/s
Arc current	109	A
Arc voltage	11.8	V
EP/EN ratio	5:1	
Contact tip to work distance	14	mm
Shielding gas flow rate	25	L/min

**Table 3:** Data of current in different stages during CMT-PADV process.

Current Polarity	Peak Current	Peak Time	Base Current	Base Time	S/C Current	S/C Time	Average Current	Cycle Time
DCEP-pulse	116.6 A	2 ms	45.8 A	1.8 ms	—	—	83.1 A	3.8 ms
DCEN-CMT	99.2 A	2.4 ms	32.2 A	5.8 ms	22.2 A	2.8 ms	44.3 A	11.0 ms

S/C stands for “short-circuiting”.

**Table 4:** Data of droplets in different current polarity periods during CMT-PADV mode.

Current Polarity	D/mm	H/mm
DCEP-pulse	1.55	1.48
DCEN-CMT	1.90	1.77

## Data Availability

Data are contained within the article.
